# Pore-scale influence of surfactants on evaporation in a porous medium

**DOI:** 10.1038/s41598-025-29925-z

**Published:** 2026-01-23

**Authors:** Ayomikun Bello, Abdolreza Kharaghani, Evangelos Tsotsas

**Affiliations:** Institute of Process Engineering, Otto von Guericke University, 39016 Magdeburg, Germany

**Keywords:** Evaporation, Porous media, Microfluidic, Surfactant, Pore network, Wettability, Engineering, Environmental sciences, Materials science

## Abstract

Despite their widespread use in controlling interfacial behavior in porous media, the pore-scale influence of surfactants on evaporation is still not well understood. This study examines how varying concentrations of sodium dodecyl sulfate (SDS) modify evaporation dynamics within a polydimethylsiloxane (PDMS) microfluidic network. Evaporation experiments were conducted using pure water and SDS solutions at 0.10 wt.% (below the critical micelle concentration, CMC), 0.23 wt.% (at CMC), 0.3 wt.%, and 0.5 wt.% (above CMC). High-resolution imaging and image analysis enabled direct quantification of liquid saturation, meniscus evolution, and contact angle behavior. At the CMC, SDS reduced surface tension from 72 mN/m to 40 mN/m, lowered capillary entry pressures, and promoted air invasion that completed evaporation 47% faster than water. Below the CMC, partial surface tension reduction improved early drainage but sustained thin films that delayed complete evaporation. The results show that tuning surfactant concentration near the CMC effectively controls pore-scale drainage and evaporation kinetics. These findings provide a foundation for developing predictive models and optimizing the design of porous materials for applications involving controlled evaporation.

## Introduction

Evaporation in porous media is a key process that affects many natural and industrial systems, such as fluid transport in porous materials, drying operations, inkjet printing, and oil recovery^1–3^. Unlike evaporation from open liquid surfaces, evaporation in porous media is controlled by capillary forces that depend on the pore structure, surface wettability, and liquid–gas interfacial tension^4^. These factors cause the evaporation process to be spatially and temporally variable, making it difficult to predict accurately. A detailed understanding of how evaporation proceeds within porous materials is therefore essential for better control and optimization in both natural and engineered systems.

Fluid displacement in porous media is commonly described by the Young-Laplace equation, which relates the capillary pressure needed for air to enter a liquid-filled pore^5,6^. Larger pores, due to their lower curvature, require less pressure to be emptied and thus tend to drain first, leading to irregular invasion fronts and complex displacement patterns^7–9^. These include patterns such as film rupture and snap-off, especially in narrow or constricted regions^7,8^. Most existing studies, however, rely on simplified models or indirect measurements that do not fully capture how the fluid interface evolves in space and time inside the pores. This limitation is especially important when studying how local wetting properties or fluid composition influence evaporation behavior^10^.

Several advances in microfluidic models of porous media have provided techniques for directly observing pore-scale dynamics^2,4,11–14^. Two-dimensional microfluidic networks with precisely etched channels allow real-time imaging of meniscus movement, film stability, and contact angle evolution. Lenormand and Zarcone (1985)^15^ first conducted experiments on two-dimensional micromodels to study the displacement of a wetting fluid (paraffin oil) by a non-wetting fluid (air) under strong capillary control. At extremely low capillary numbers $(\sim 10^{-8} - 10^{-7})$, they observed dendritic invasion patterns characteristic of invasion percolation. They also found that as the capillary number increased, saturation decreased due to wetting fluid escape along pore roughness, while the fractal dimension of the displacement patterns remained consistent.

Primkulov et al.^16^ introduced a quasistatic invasion percolation framework capable of simulating displacement dynamics across a full range of contact angles, from strong drainage to strong imbibition. Building on this, Jahanbaksh et al.^17^ investigated how spatial variations in surface wettability influence two-phase fluid displacement in an otherwise homogeneous porous medium. Using a quasi-3D pore-scale model, they showed that heterogeneity in wettability significantly alters fluid invasion patterns, interface evolution, saturation behavior, and displacement efficiency. Dollet et al.^18^ investigated evaporation in PDMS microchannels under controlled dry conditions to isolate the influence of channel geometry. They showed that evaporation occurred faster in narrower channels and thinner layers because the liquid–air interface was more affected by the surrounding solid boundaries. This highlighted how geometric confinement can significantly impact local evaporation dynamics. Furthermore, Rahimi et al.^19^ developed a pore network model to study evaporation from porous substrates where surface pores interact with evaporating droplets. Their work provided a simplified framework to understand how pore connectivity and liquid supply at the surface influence evaporation behavior in porous systems.

Karapetsas et al.^20^ developed a model for evaporating thin droplets containing insoluble surfactants and non-interacting particles. Their analysis revealed that surfactants influence droplet evaporation through competing mechanisms, which increase evaporation by enlarging the interfacial area and suppressing evaporation, which arise from reduced effective surface area and altered flow dynamics. Similarly, Farhadi and Bazargan^21^ investigated how thermally and surfactant-induced flows shape internal fluid dynamics in evaporating sessile droplets with pinned contact lines. Their results showed that surfactant accumulation near the droplet apex and temperature maxima near the edge create dual surface tension minima. This generates a surface tension peak between them, driving a double-vortex circulation pattern inside the droplet.

Furthermore, Marin et al.^22^ investigated how different surfactants influence internal flow patterns in evaporating water droplets. In droplets without surfactants, they observed a flow moving from the droplet edge toward the center, which strengthened as evaporation continued. When Tween 80, a non-ionic surfactant, was added above its critical micelle concentration (CMC), the droplet surface became less mobile, suppressing both surface and internal circulation. In contrast, sodium dodecyl sulfate (SDS), an anionic surfactant, at concentrations above its CMC, increased flow velocities near the contact line. These differences result from the molecular structure and adsorption behavior of each surfactant, which influence surface mobility and can either suppress or enhance particle deposition during evaporation.

However, without direct pore-scale observation, the mechanisms by which surfactants influence meniscus curvature, contact angle hysteresis, and film coalescence remain speculative. In our experiments, we address this knowledge gap by using a PDMS-based microfluidic network composed of a 5 × 5 array of square pores (1 mm side length) connected by throats ranging from 0.14 to 0.94 mm. This design produces a broad distribution of capillary entry thresholds, replicating the heterogeneity of natural porous media while enabling direct optical visualization.

Previous studies^7,11,12,23,24^ have emphasized the importance of cooperative pore-scale phenomena in controlling macroscopic drying behavior. These include liquid cluster fragmentation, meniscus movement and capillary-driven flow redistribution. Wu and Zhao^12^ showed that the positioning of the meniscus within the liquid groups dynamically governs local flow rates, with flow correlations exhibiting power-law scaling that depends on the saturation levels and the position of the meniscus. In Wu et al.^7^, a dynamic pore network model incorporating capillary valves and both continuous and discontinuous corner films was developed and validated against micromodel experiments. The study showed that although continuous corner films keep high evaporation rates, they can break down due to gas invasion or unstable capillary forces.

Although these works have clarified the influence of network geometry and structural heterogeneity on evaporation dynamics, the role of surfactant-modified interfacial properties in governing pore-scale behavior remains unstudied. Therefore, in this study, we investigate how variations in the surfactant mass fraction influence air invasion patterns and evaporation dynamics within a PDMS microfluidic pore network. We conducted high-resolution imaging of the evaporation process at surfactant mass fractions below, at, and above the critical micelle concentration (CMC), quantified the evolution of saturation over time, and extracted average contact angles during intermediate stages of evaporation.

## Materials and methodology

### Microfluidic pore network design

**Fig. 1:**
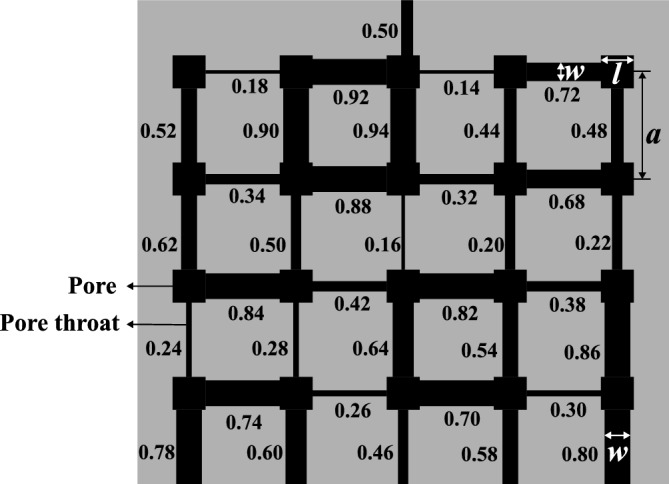
Schematic of the PDMS microfluidic pore network with graded throat widths (values labeled in mm). The layout is schematic, and *a* and *l* are not drawn to scale.

To investigate the influence of surfactants on evaporation, we conducted a series of pore-scale experiments using a microfluidic pore network. The experiments were performed with a PDMS-based pore network which mimics a porous medium and consists of regularly arranged square pores connected by rectangular throats. Each pore has a uniform side length of 1 mm, while the center-to-center spacing between adjacent pores is 2 mm, defining the fixed throat length of 1 mm. The throats, which control the flow constriction between pores, have varying widths ranging from 0.14 mm to 0.94 mm. The design is constrained to a 5 × 5 pore array. The layout is illustrated in Fig. 1, where the labeled numbers represent individual throat widths in millimeters. One side of the network includes a 0.5 mm wide channel connected to the central pore. The opposite side is fully open to the ambient environment, facilitating slow evaporation. The remaining two sides are sealed, preventing fluid exchange. All features in the network, including both pores and throats, have a constant depth of 0.1 mm perpendicular to the plane of the image.

### Fluids

The fluids used in this study include distilled water and aqueous solutions of an anionic surfactant, Sodium Dodecyl Sulfonate (SDS) *(Carl Roth GmbH, Germany)*, prepared at four different mass fractions. The selected mass fractions were 0.10 wt.%, 0.23 wt.%, 0.30 wt.%, and 0.50 wt.%. The rationale behind these selected concentrations lies in their relation to the critical micelle concentration (CMC) of the surfactant. The CMC is the concentration beyond which surfactant molecules begin to aggregate in the bulk liquid. For SDS, the CMC is approximately 0.23 wt.%. By selecting mass fractions below (0.10 wt.%), at (0.23 wt.%), and above (0.30 wt.% and 0.50 wt.%) this critical point, the experiments investigate changes in interfacial properties, such as surface tension and wettability, affect capillary flow behavior during evaporation.

### Evaporation experiments

Before every experiment, the microfluidic pore network was thoroughly cleaned to ensure removal of any residual contaminants that could interfere with fluid behavior during evaporation. The cleaning process began with flushing the entire network with a mixture of isopropanol and acetone, to remove any residual impurities. Following this, the network was rinsed with deionized water to eliminate any residual alcohol. The pore network was then allowed to dry completely. Once dry, the microfluidic device was placed in the test liquid contained in a cylinder. To achieve the saturation of the pore space, the setup was transferred into a vacuum chamber *(Binder GmbH, Germany)*. As such, trapped air bubbles within the network were evacuated, allowing the liquid to infiltrate the pores and throats.

**Fig. 2:**
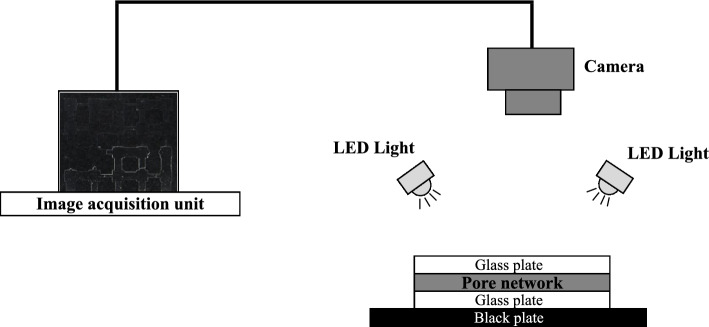
Schematic diagram of the experimental setup during evaporation tests. The microfluidic pore network rests on a matte black plate to reduce reflection and enhance contrast. A vertically mounted high-resolution camera with macro lens captures images automatically, while uniform illumination is provided by a four-way white LED lighting system.

Following saturation, the microfluidic chip was removed from the chamber and positioned horizontally on a matte black plate that minimized light reflection and aided contrast during imaging. The experiments were conducted under controlled ambient conditions, with the temperature maintained at 25±$2^\circ$C and relative humidity stabilized at 22±2%. The experimental setup is illustrated in Fig. 2.

The progression of liquid evaporation and redistribution within the pore network was recorded using a high-resolution digital camera *(Nikon D810, Japan)* equipped with a macro lens *(AF-S VR Micro-Nikkor 105 mm f/2.8G IF-ED, Japan)*. The camera was mounted vertically and operated via computer control using digiCamControl v2.1.7 software, which enabled automated image capture at 1-minute intervals throughout the evaporation process. To ensure uniform and high-quality illumination across the pore network, a four-way white LED lighting system *(CCS Inc., Japan)* was employed. This setup provided bright, stable lighting conditions that enhanced the contrast between gas and liquid phases in the captured images. The resulting image sequence was processed using a custom-built image analysis algorithm developed in Python. This algorithm enabled the extraction of key parameters such as the distribution of the liquid phase and the evolution of its saturation over time, forming the basis for quantitative analysis of the evaporation dynamics in our work.

### Image processing

**Fig. 3:**
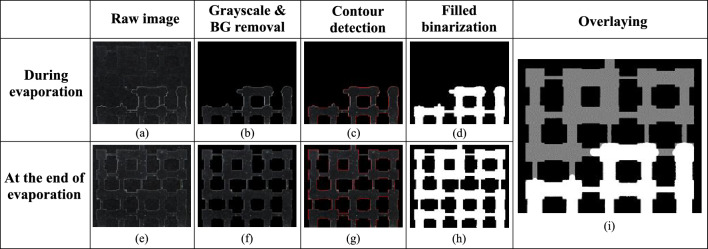
Image processing algorithm. (**a**) Raw image during evaporation; (**b**) grayscale image after background removal during evaporation; (**c**) contour detection of pores during evaporation; (**d**) information on positions of solid pixels (black) and gas pixels (white) for the image during evaporation; (**e**) raw image at the end of evaporation; (**f**) grayscale image after background removal at the end of evaporation; (**g**) contour detection of pores at the end of evaporation; (**h**) information on positions of solid pixels (black) and gas pixels (white) for the image at the end of evaporation; (**i**) overlaid image showing information on positions of solid pixels (black), gas pixels (white) and liquid pixels (grey) during evaporation.

To quantitatively analyze fluid distribution and the evaporation front within the pore network, we implemented an image processing algorithm using Python and OpenCV, and then used *ImageJ* software for contact angle analysis. Each raw image was converted to grayscale to reduce computational complexity while retaining the necessary contrast between filled and unfilled pore network (Fig. 3). To remove noise and enhance the definition of pore boundaries, morphological operations were applied using a structuring sequence which consisted of the opening (erosion followed by dilation), to eliminate small white artifacts (isolated pixels or specks) and smoothen the outer boundaries of larger pore regions; and the closing (dilation followed by erosion), which filled minor black voids within white pore areas, improving the continuity and completeness of pore shapes. These operations helped refine the binary mask, making the subsequent detection of pore contours more accurate and reliable. Afterwards, the contours were extracted using OpenCV’s cv2.findContours() function with the RETR_TREE retrieval mode, which detects both outer and inner contours. Following that, two output images were generated:

- An annotated version of the original color image, with red contour lines overlaid to visually highlight pore boundaries for qualitative assessment.
- A filled binary image where all identified pore contours were drawn and filled with white to offer a clear mask of the pore regions that have been invaded by air.

However, this does not still give the final picture and cannot help determine the pore areas with liquid and air, since not all the black represent liquid as some part of the pore network is still solid. Therefore, to track the evolution of fluid distribution over time, we overlaid binary images taken at intermediate time steps during the evaporation with the final image taken after evaporation is complete, that is, when the pore network has only solid and gas. Both images were thresholded at a value of 127 to create Boolean masks. As such, the overlay image was generated where gray, white and black represent liquid, air and solid respectively. This overlap quantifies how much of the final pore space was already invaded during the drying process and can be calculated in percentage as the ratio of liquid pixels to the total number of liquid and gas pixels or the initial gray pixels.

For further analysis of wettability effects, we used *ImageJ* software equipped with the DropSnake plugin to measure contact angles^25,26^. This plugin employs active contour modeling (B-spline snakes) to fit the shape of the menisci at the interface with high precision.

## Results and discussion

### Evaporation patterns for the fluids

**Fig. 4:**
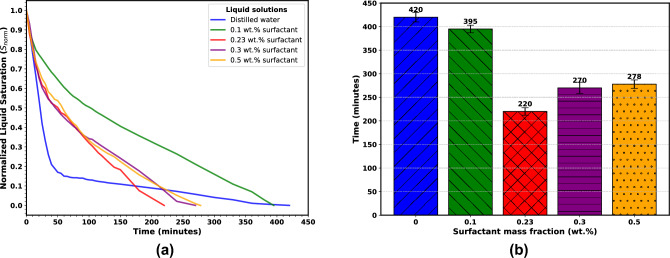
Evaporation patterns. (**a**) Evaporation curves (normalized liquid saturation vs. time) for pure water and SDS solutions; (**b**) Total evaporation time for water and SDS solutions at various concentrations.

Figure 4a presents the evolution of the normalized liquid saturation, $S_{\textrm{norm}}(t)$, within the microfluidic pore network for distilled water and the aqueous surfactant solutions. Here $S_{\textrm{norm}}(t)$ is defined as the global liquid saturation at time $t$ divided by the initial network saturation, $S(t=0)$

1 $$\begin{aligned} S_{\textrm{norm}}(t) = \frac{S(t)}{S(t=0)} \end{aligned}$$

As can be seen, at early times, all fluids exhibit a rapid decrease in $S_{\text {norm}}$, reflecting the evaporation of the largest throats by capillary invasion once the local entry pressure threshold is exceeded. Distilled water (blue curve) shows the steepest initial decline, with $S_{\text {norm}}$ dropping below 0.2 within the first 50 min. This behavior can be attributed to the high surface tension between air and water, which generates larger capillary pressures at a given throat radius, thus promoting rapid air fingering through the network’s widest constrictions^27^.

**Fig. 5:**
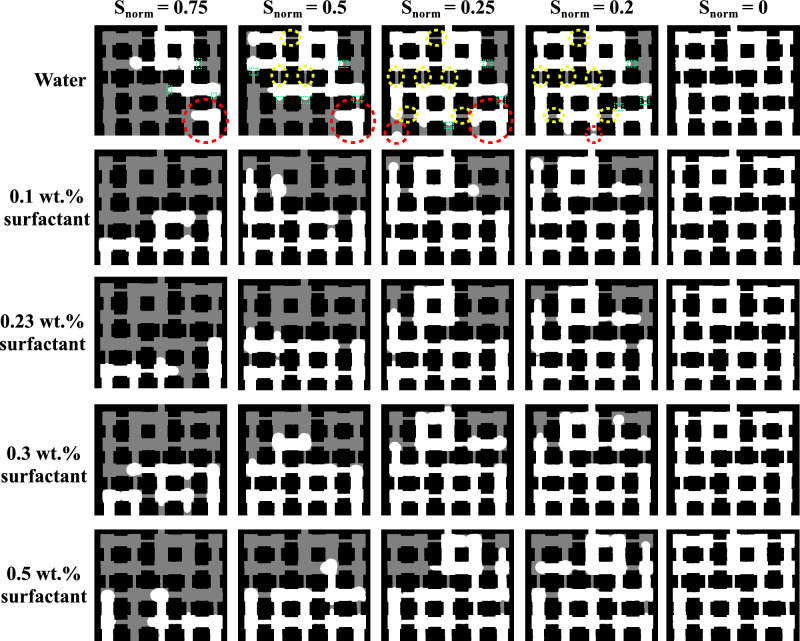
Pore-scale saturation front images during evaporation for water and SDS solutions. Red dashed circle show the isolated pocket of air; Green dashed boxes show the curved menisci; Yellow dashed circles show the disconnected liquid islands.

At CMC (red curve), it can be seen that the 0.23 wt.% solution evaporates faster than water, reaching zero saturation by  220 min (Fig. 4b). A plausible explanation is that at this concentration, dynamic interfacial effects due to the influence of surfactant, lead to transient increases in local capillary pressure heterogeneity, causing preferential invasion through slightly larger throats and increasing overall evaporation once an invasion path percolates. These interfacial changes reduce the capillary entry pressure in the porous medium, enabling air to invade pores at higher liquid saturations^28,29^. The pore-scale images of saturation evolution in the pore network is shown in Fig. 5 to provide further explanation on this.

From Fig. 4, we have seen that water, with its high surface tension (Table 1), exhibits the slowest evaporation, approximately 420 minutes to reach complete evaporation. An initial steep drop in $S_{\text {norm}}$ (within the first hour) corresponds to rapid evaporation from surface pores; followed by a continuous, more gradual decline as the remaining liquid films persist under strong capillary retention and limited connectivity^30,31^. In Fig. 5, without surfactant in the liquid phase, we see that air enters the largest pore throats, which creates small, isolated pocket of air (red dashed circle) surrounded by a continuous liquid network. The menisci (the curved surfaces between air and liquid) are very curved and get trapped in narrow throats (green dashed boxes). When $S_{\text {norm}}$ decreases, we start seeing many small, separate islands of liquid (yellow dashed circles). This shows that the liquid is poorly connected, and that the air moves through in a fingering pattern, which confirms that water allows air to easily enter and break the liquid into separate islands.

When we compare different surfactant mass fractions, we find that low mass fraction (0.1 wt.%) produce results which are different from pure water, while higher mass fractions above the CMC show only small differences.

For the 0.1 wt.%, which is below the CMC, the total evaporation time decreases to about 395 minutes, and air penetrates more pores than for pure water, so evaporation is generally faster. At $S_{\textrm{norm}} \approx 0.5$, many air pathways are well connected, and there are fewer liquid islands. This shows that the surfactant lowers the pressure needed for air to enter and helps air move through narrow throats. Also, the menisci are flatter than with pure water, meaning the liquid is more wetting (lower contact angle), as later shown in Fig. 11 and discussed in Section 3.2.

At the CMC, the air invasion pathways are visibly connected across the network. The menisci are broader, and pores are connected by liquid bridges. By $S_{\textrm{norm}} \approx 0.5$, this connected structure is even more visible. The menisci have largely merged into fewer, larger interfaces, leading to faster evaporation.

When the mass fraction is above the CMC, evaporation becomes slower again, extending total evaporation to around 270 and 278 minutes. The images look very similar to the 0.23% case, though the menisci appear flattest at these higher concentrations. This may be because too much surfactant induces micelle formation, which can block vapor movement and reduce air entry^32,33^.

In Fig. 4a, we see that the surfactant solutions even show a slower reduction in evaporation during the early stages. This is attributed to a reduced surface tension, typically from $72\,\text {mN/m}$ (water) to the range of $32 - 40\,\text {mN/m}$ for SDS surfactants^34^, reducing the Laplace pressure.

**Fig. 6:**
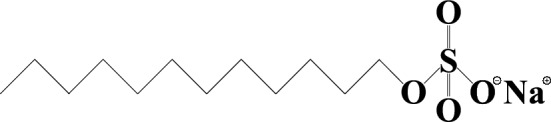
Molecular structure of SDS surfactant.

SDS is a classic anionic surfactant with its sulfate head strongly hydrophilic and negatively charged, while its 12 carbon alkyl tail is hydrophobic (Fig. 6).

In aqueous solutions, SDS molecules accumulate at the air water interface, where they reduce surface tension. When the concentration exceeds the critical micelle concentration (CMC), the molecules also self-assemble into micelles. These molecular interactions influence how water molecules transition into the vapor phase. Because SDS is non-volatile^35^, it slightly lowers the vapor pressure of water through a colligative (Raoult’s-law) effect, while the interfacial SDS layer further modifies the evaporation behavior by restricting the escape of water molecules, leading to a reduced evaporation rate compared to other cases with surfactant mass fraction below the CMC^36,37^. We note that SDS does not form a rigid film like an insoluble monolayer. Instead it creates a fluid, loosely packed surface layer and engages in dynamic exchange with bulk water^38^.

We hypothesize that surfactants could enhance evaporation by reducing the energy barrier for molecules to escape from the liquid surface. Table 1 shows the surface tensions of the surfactant mass fractions used in this work.

**Table 1:** Surface tension values at different surfactant mass fractions.

Surfactant mass fraction (wt.%)	Surface tension (mN/m)
Water	72.01
0.1	62.56
0.23	39.95
0.3	36.83
0.5	32.16

Reduced surface tension directly decreases the capillary entry pressure, $P_c$, needed for air to enter pores and throats. For a throat of constant width:

2 $$\begin{aligned} \frac{P_c^{\text {s}}}{P_c^{\text {w}}} = \frac{\sigma _{\text {s}}}{\sigma _{\text {w}}} \end{aligned}$$

Given that the surface tension of the $0.1\%$ surfactant solution, $\sigma _s = 62.56\,\text {mN/m}$, compared to that of water, $\sigma _w = 72.01\,\text {mN/m}$, the resulting $P_c$ decreases by roughly $13\%$. This reduction in capillary pressure enhances the air invasion velocity, $v \propto \frac{1}{P_c}$, thereby accelerating liquid displacement.

Without surfactant, the surface tension is relatively high, so the capillary pressure required to enter a square pore through a rectangular throat is:

3 $$\begin{aligned} P_c = 2 \, \sigma _w \, \cos \theta \left( \frac{1}{w} + \frac{1}{h} \right) \end{aligned}$$

where $\sigma _w$ is the surface tension of water, $\theta$ is the contact angle, and $w$ and $h$ are the width and height of the rectangular throat, respectively.

Assuming an ideal wetting situation with a near-zero receding contact angle ($\theta \approx 0^\circ$, thus $\cos \theta \approx 1$), the high $\sigma$ means that narrow rectangular throats become significant barriers. For a throat of width $w = 50~\mu \textrm{m}$ and height $h = 50~\mu \textrm{m}$, $P_c \approx 5.76 \times 10^3~\textrm{Pa}$. When SDS is added at its critical micelle concentration, the surface tension decreases ($\sigma _{\textrm{s}} \approx 39.95~\mathrm {mN/m}$)^34^, lowering the capillary entry pressure for the same throat to $P_c \approx 2.52 \times 10^3~\textrm{Pa}$.

Such high pressures, in the case of water, delay air from entering small pores, resulting in a distinct two-step evaporation curve, that is, an initial fast drainage of larger pores followed by a highly diffusion-limited stage when the remaining liquid is trapped in constricted spaces^7^. With surfactant, the lower capillary threshold allows air to invade earlier, so pores start evaporating at lower pressures.

At surfactant mass fractions below the CMC, the reduction in surface tension is moderate, leading to a slight decrease in capillary entry pressure compared to pure water. Because the capillary entry pressure in a porous medium is directly proportional to surface tension, this reduction lowers the pressure required for air invasion. Consequently, the invasion of air becomes easier than with pure water, although the effect remains limited since surface tension is only partially reduced below the CMC.

At mass fractions near or above the CMC, further addition of surfactant no longer significantly reduces the static surface tension, since the interface is saturated. Consequently, the capillary entry pressure remains effectively constant beyond this point. Therefore, variations in surfactant mass fraction may strongly affect the air invasion process primarily below the CMC, while above this threshold the system reaches a point where further additions have no significant effect.

It is important to note that the dominant effect of SDS, and perhaps other surfactant types, on evaporation is controlled by surface effects. When SDS is added below its CMC, it significantly reduces the surface tension of water. This reduction in surface tension changes how the liquid distributes itself within the porous network, allowing it to spread more easily and create a larger liquid air interface. Since evaporation primarily occurs at this interface, an increase in interfacial area enhances the overall evaporation rate.

In Kwieciski et al.^10^, the results showed that even very dilute SDS solutions ($\sim 0.025\text {-}0.1~\text {CMC}$) produced noticeably flatter droplets. The initial contact angle decreased with increasing SDS concentration, and the droplet developed a local contact angle minimum up to $\sim 35^{\circ }$ lower than that of pure water. This spreading increases the liquid-air interface and enhances the overall evaporation rate.

### Role of wettability on evaporation

The evaporation behavior observed in our pore network can be understood through the physics of primary drainage, where air displaces water or the surfactant solution. During this process, air enters the pores following invasion percolation principles, as it first invades the largest throats, which require the lowest capillary pressure to overcome. As a result, the air pathways form an irregular but predictable pattern that reflects the balance between pore geometry and capillary resistance, as governed by the Young-Laplace relation.

In our rectangular pore network, the capillary entry pressure driving air invasion has been given in Equation 3. Since the pore network has uniform height $h$ but varying widths $w$, the second term remains constant while the first term varies with $w$, making narrow throats more significant barriers to air invasion. Thus, the pressure needed to advance the air phase, and hence evaporate the liquid, depends sensitively on both the local geometry of the pore and the value of the contact angle.

Since the height is constant across the network, the analysis can focus primarily on variations in width. The estimated entry pressures scale inversely with *w*, meaning that wider throats drain first because they have lower capillary entry thresholds. This agrees with the experimental observation that air preferentially invades wider channels before moving into adjacent pores.

The pore scale images in our experiments reveal different meniscus shapes and suggest the existence of two invasion modes, the bursting and merging modes as earlier noted in Wu et al.^14^.

**Fig. 7:**
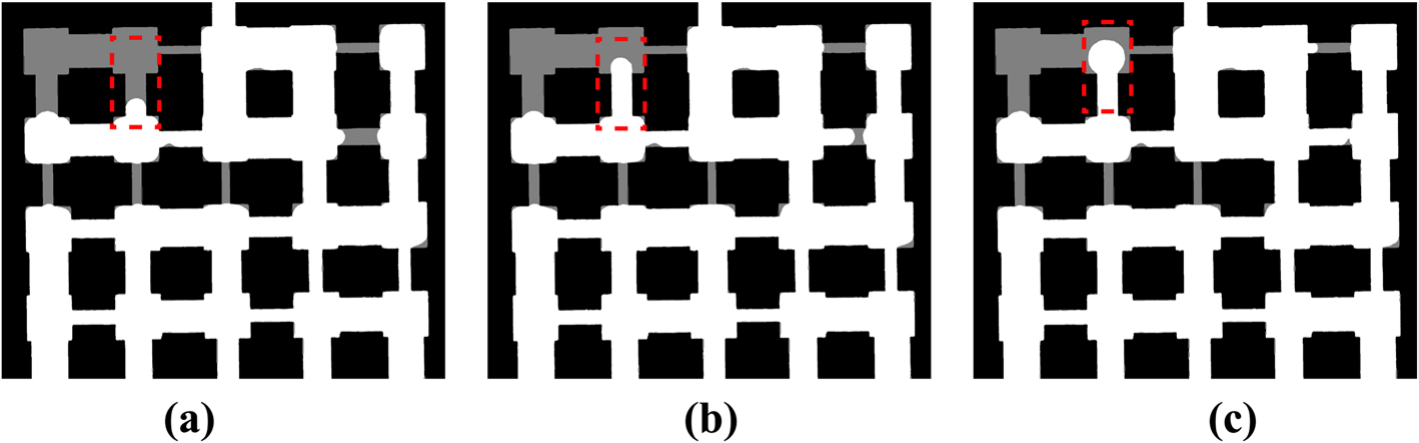
Bursting invasion sequence. (**a**) Early stage; (**b**) intermediate stage; (**c**) final stage.

Figure 7 shows a sequence of the air - water meniscus during the bursting invasion. In Fig. 7a, the meniscus is concave against the pore wall. In Fig. 7b, the meniscus becomes slightly less curved as the contact angle increases. These images show that the meniscus is progressively losing its capillary hold until the pore suddenly bursts open to air in Fig. 7c.

Bursting invasion occurs when an air-water interface pinned at a narrow throat is forced into a larger pore. The invading air remains *“stuck”* at the throat until its pressure exceeds the capillary threshold (the burst pressure). In other words, the pore acts as a capillary valve, such that the meniscus stops at the expansion until the pressure builds to a critical value. Once that threshold is reached, the meniscus snaps forward and the air floods the pore, which is the bursting event. In our experiment, the meniscus is initially pinned (Fig. 7a,b) and then bursts into the pore in Fig. 7c when the driving pressure just overcomes the capillary barrier.

**Fig. 8:**
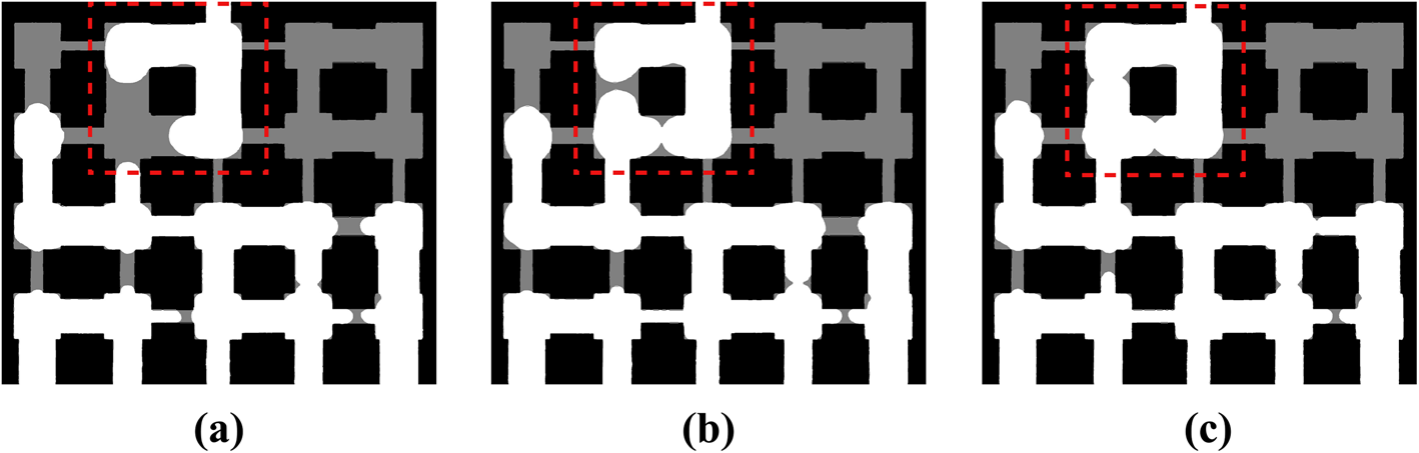
Merging invasion sequence. (**a**) Two separate menisci in adjacent throats approaching a common pore; (**b**) menisci touching and coalescing at the pore entrance; (**c**) resulting single merged meniscus rapidly advancing into the pore.

In the case of merging invasion, two wetting-phase menisci in neighboring throats advance together and meet at a pore entrance, coalescing into one larger meniscus. Figure 8 illustrates this. Initially two separate menisci (Fig. 8a) approach the shared pore, then touch, and finally merge into a single meniscus that invades the pore.

**Fig. 9:**
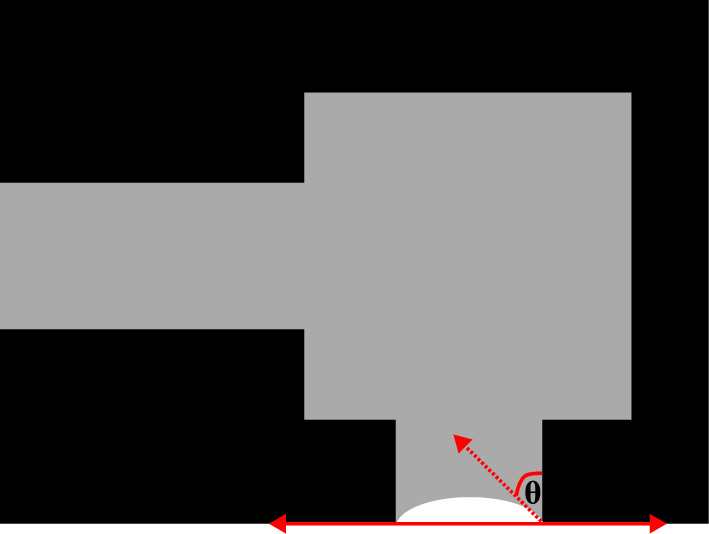
Contact angle measurement.

The wettability of a fluid within the porous media fundamentally governs fluid distribution and phase behavior during evaporation, as quantified by the contact angle at the interface. In this study, contact angles were measured at $S_{\text {norm}} = 0.5$, corresponding to the mid-stage of evaporation, where a defined meniscus is observable. The contact angle is determined as shown in Fig. 9.

**Fig. 10:**
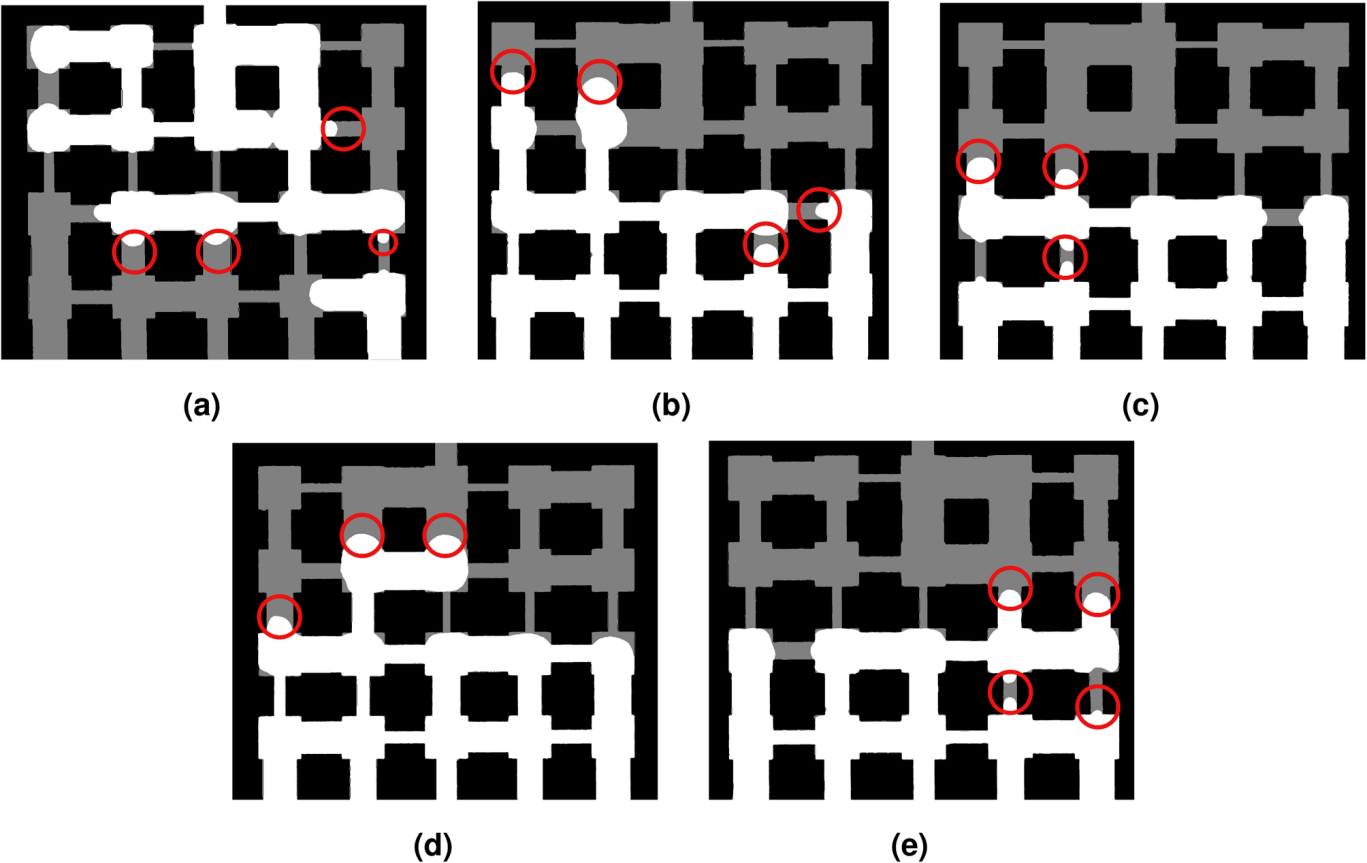
The advancing menisci used for contact angle measurements of different fluids when the normalized liquid saturation is 0.5. (**a**) Pure water; (**b**) 0.10 wt.% SDS; (**c**) 0.23 wt.% SDS; (**d**) 0.30 wt.% SDS; (**e**) 0.50 wt.% SDS.

**Fig. 11:**
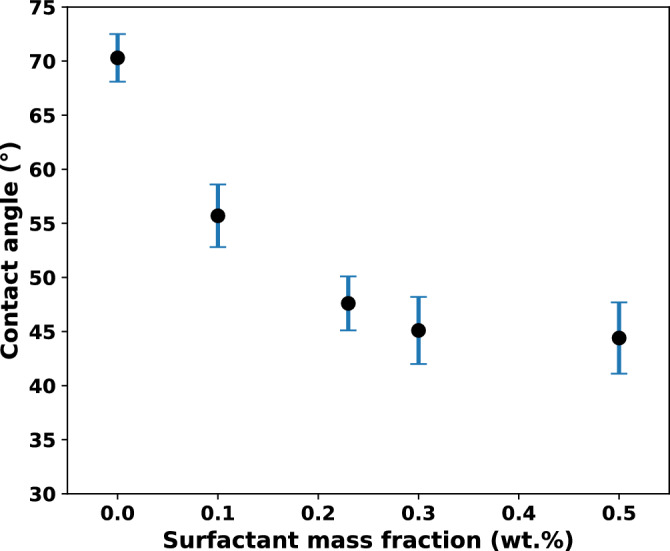
Contact angle averaged over all the points of the evaporation front in the meniscus in the model and averaged over three measurement trials. The error bars show standard errors of the mean.

To evaluate the contact angles, all identifiable menisci within the pore throats at $S_{\text {norm}} = 0.5$ were measured, as illustrated in Fig. 10. For each liquid, the contact angles measured from three experimental trials were averaged, and the mean values are shown in Fig. 11. The total number of meniscus points analyzed was 15, 18, 12, 13, and 14 for water, 0.1 wt.% SDS, 0.23 wt.% SDS, 0.3 wt.% SDS, and 0.5 wt.% SDS, respectively.

In Fig. 11, water shows a relatively high meniscus contact angle compared to the other fluids, and the addition of SDS causes a steady decrease in contact angle.

When the meniscus contact angle is less than 90°, it curves inward toward the air phase, allowing air to enter even small pores more easily^39^. Figure 11 illustrates that adding SDS lowers the contact angle and increases the wettability of the surface. This explains why, in water, the air phase moves slowly and unevenly, creating unstable, finger-like invasion patterns. Large contact angles indicate poor wetting of the solid surface, leading to irregular front advancement^40^.

As shown in Fig. 11, at SDS mass fractions between 0.10 and 0.23 wt.%, the average contact angle falls below 90°, meaning the pore surfaces can now be wetted more easily. The surfactant reduces interfacial tension, which lowers the capillary pressure needed to invade small pores. As contact angle decreases below 90°, capillary pressure drops, allowing air to move more smoothly into smaller pores. Consequently, at low SDS concentrations, the meniscus shape supports uniform air entry even through narrow throats, resulting in the smooth invasion fronts shown in Fig. 5. In contrast, water, with its high contact angle, produces isolated air pockets and finger-like invasion patterns.

When the surfactant mass fraction increases beyond the CMC, the average contact angle no longer changes significantly (Fig. 11). Beyond this point, adding more surfactant no longer improves wettability significantly. This agrees with previous studies^10,41^ showing that higher SDS concentrations could even lead to the reappearance of fingering patterns and a reduction in evaporation rate.

Similar trends have been reported by Kwieciski et al.^10^, where increasing SDS concentration reduced the initial contact angle because of lower surface tension. They also observed that droplets with low SDS concentrations (< 0.1 CMC) reached much smaller contact angles of up to 35° lower, than droplets at 1 CMC.

## Conclusions

In this study, we investigated how varying mass fractions of sodium dodecyl sulfate (SDS) influence pore-scale evaporation dynamics in a PDMS microfluidic porous network. Using image-based measurements and saturation curves, we demonstrated how surfactants alter surface tension, wettability, and invasion modes, to influence evaporation. The key findings are summarized as follows:

- At the CMC, SDS achieves an optimal balance between surface tension and wettability. This allows air to enter pores more easily, forming a smooth and connected drying front. As a result, the total evaporation time is reduced by almost half compared to pure water.
- The addition of surfactant lowers the contact angle, which means the liquid spreads more easily. For water, the contact angle is high and air moves unevenly through the pore network. With surfactant, the contact angle becomes smaller, allowing air to pass smoothly through small pores and speeding up evaporation.
- The invasion dynamics evolve from a fingering-dominated regime, typical of pure water, to a cooperative drainage mode in the presence of surfactant, which promotes liquid redistribution and more uniform evaporation.
- The capillary entry pressure decreases proportionally with surface tension reduction up to the CMC, beyond which further surfactant addition produces negligible change. This indicates that interfacial saturation defines a limiting condition for surfactant effectiveness.
- These findings demonstrate that precise control of surfactant concentration provides an effective means to regulate pore-scale evaporation kinetics. Future work could explore the effects of dynamic adsorption, and varying pore geometries to develop more accurate models of surfactant evaporation in porous systems.

## Data Availability

The datasets used and/or analyzed during the current study are available from the corresponding author upon reasonable request.
